# Impact of early child-parent bonding on violence in patients with schizophrenia

**DOI:** 10.1192/j.eurpsy.2025.397

**Published:** 2025-08-26

**Authors:** X. Ling, S. Wang, N. Li, Q. Zhang, H. Li

**Affiliations:** 1 Shanghai Key Lab of Forensic Medicine, Key Lab of Forensic Science, Ministry of Justice, Shanghai Forensic Service Platform, Academy of Forensic Science; 2 Department of Forensic Medicine, School of Basic Medical Sciences, Fudan University; 3 Shanghai Hongkou Mental Health Center, Mental Health Center Affiliated to Shanghai University School of Medicine, Shanghai, China

## Abstract

**Introduction:**

Violence is a major global health concern among patients with schizophrenia. However, the triggers of violent behavior remain unclear. In previous studies, familial risk factors are believed to be associated with mental disorders and violence. The relationship between parental bonding or childhood adversity and psychopathologic behavior (such as violence) has rarely been evaluated.

**Objectives:**

The study aimed to explore the relationship between violent behavior and childhood experience and to determine the role of the early child-parent bond in violence risk in patients with schizophrenia.

**Methods:**

The study enrolled 287 patients with schizophrenia and 100 healthy controls. Patients were divided into 3 groups: patients with homicidal history (Group A), patients with violent behavior and without homicidal history (Group B) and patients without violent behavior (Group C). Childhood trauma questionnaire (CTQ), parental bonding instrument (PBI) and modified overt aggression scale (MOAS) were used to explore the violent behavior and childhood experience. All individuals participated voluntarily and provided informed consent. This study was approved by the ethics committee of the Academy of Forensic Science.

**Results:**

The findings indicated the proportion of males to be higher in the patient groups than in the healthy controls, especially in the group with homicidal history. Patients had a significantly higher prevalence of sexual abuse, emotional abuse and emotional neglect than the healthy controls. The emotional abuse and emotional neglect were found to be positively and negatively related to MOAS scores. Maternal over protection was found to be negatively related to the MOAS scores. On the CTQ subscales, emotional neglect was significantly associated with violence risk (OR=1.13, 95% CI=1.04–1.22). On the PBI subscales, maternal and paternal care (0.84, 0.74–0.94 and 1.30, 1.13–1.49) and over protection (1.18, 1.07–1.29 and 0.87, 0.81-0.95) were found to be significantly associated with violence risk. Maternal and paternal over protection were significantly associated with homicide risk (0.87, 0.78-0.97 and 1.10, 1.01-1.20).

**Conclusions:**

The schizophrenia patients with violence might suffer lower paternal care and emotional abuse during the childhood. In terms of violence in schizophrenia patients, paternal over protection and maternal care might be a protective factor and emotional neglect, maternal over protection and paternal care might be a risk factor. In terms of homicide in schizophrenia patients, paternal over protection might be a risk factor and maternal over protection might be a protective factor. Therefore, childhood trauma and parental care and over protection could be a potential reference indicator for assessing violence risk in patients with schizophrenia.

**Disclosure of Interest:**

X. Ling: None Declared, S. Wang: None Declared, N. Li: None Declared, Q. Zhang: None Declared, H. Li Grant / Research support from: This study was supported by National Key R & D Program of China [grant number 2022YFC3302001], National Natural Science Foundation of China [grant number 81801881], Science and Technology Committee of Shanghai Municipality [grant numbers 20DZ1200300, 21DZ2270800, 19DZ2292700].

